# Early life exposure to ethinylestradiol enhances subsequent responses to environmental estrogens measured in a novel transgenic zebrafish

**DOI:** 10.1038/s41598-018-20922-z

**Published:** 2018-02-09

**Authors:** Jon M. Green, Anke Lange, Aaron Scott, Maciej Trznadel, Htoo Aung Wai, Aya Takesono, A. Ross Brown, Stewart F. Owen, Tetsuhiro Kudoh, Charles R. Tyler

**Affiliations:** 1 Biosciences, College of Life and Environmental Sciences, University of Exeter, Geoffrey Pope, Stocker Road, Exeter, Devon, EX4 4QD UK; 2 AstraZeneca, Global Environment, Alderley Park, Macclesfield, Cheshire, SK10 4TF UK

## Abstract

Estrogen plays fundamental roles in a range of developmental processes and exposure to estrogen mimicking chemicals has been associated with various adverse health effects in both wildlife and human populations. Estrogenic chemicals are found commonly as mixtures in the environment and can have additive effects, however risk analysis is typically conducted for single-chemicals with little, or no, consideration given for an animal’s exposure history. Here we developed a transgenic zebrafish with a photoconvertable fluorophore (Kaede, green to red on UV light exposure) in a skin pigment-free mutant element (ERE)-Kaede-Casper model and applied it to quantify tissue-specific fluorescence biosensor responses for combinations of estrogen exposures during early life using fluorescence microscopy and image analysis. We identify windows of tissue-specific sensitivity to ethinylestradiol (EE2) for exposure during early-life (0–5 dpf) and illustrate that exposure to estrogen (EE2) during 0–48 hpf enhances responsiveness (sensitivity) to different environmental estrogens (EE2, genistein and bisphenol A) for subsequent exposures during development. Our findings illustrate the importance of an organism’s stage of development and estrogen exposure history for assessments on, and possible health risks associated with, estrogen exposure.

## Introduction

Exposure to endocrine disrupting chemicals (EDCs) is linked with a range of adverse health disorders and further understanding of EDCs effects is crucial for safe-guarding long-term human and environmental health^1,2^. Many EDCs with estrogenic activity enter the aquatic environment via waste discharges and there are associations between exposures to specific environmental estrogens (e.g. the contraceptive estrogen, 17α-ethinylestradiol, EE2) and adverse health effects in individual fish^3,4^ and fish populations^5,6^. Laboratory based studies on fish evidence associations between various environmental estrogens and feminization of males^3,7^ and alteration of sexual behavior^8^. In mammals too, exposure to environmental estrogens has been associated with decreases in semen quality/sperm count^9^, heart disease and diabetes^10^. Exposure to estrogenic chemicals during early life-stages in both mammals and fish has received much recent attention with reports of significant adverse physical and behavioral effects^11–13^.

Exposures to estrogens in the natural environment occur predominantly as mixtures and studies both *in vitro* (e.g reporter gene assays^14–16^) and *in vivo* (fish^17^, mammals^18,19^) have illustrated the capacity for additive (and greater than additive) effects. Studies on chemical mixtures have suggested enhanced tissue-specific effects may occur, for example as seen for responses to EDC mixtures in mammary gland development in rats^18,19^. Effects analysis for exposures representative of real world scenarios is therefore complicated by mixture permutations, chemical interactions and tissue-specific responses.

There are two nuclear ER subtypes in mammals, Esr1 and Esr2^20^, and three in zebrafish, Esr1, Esr2a and Esr2b^21,22^. Other ER subtypes include membrane ERs (mERs), estrogen-related receptors (ERRs)^23–25^ and interaction of ERs with estrogen response elements (EREs) and their downstream expression sequences can be regulated by various co-factors^26,27^. The expression of ER subtypes in organs and tissues can vary during life, influencing the physiological targets and subsequent downstream effects^28–31^. Exposure to estrogenic chemicals during early life has been shown to increase expression of ERs with tissue-specific targeting for these chemicals^31^. This effect of sensitization and increased responsiveness has been shown to persist even after a prolonged phase of depuration^3^.

Estrogen responsive transgenic zebrafish models have been developed with an estrogen response element (ERE) transgene^32–35^ or brain-specific *cyp19a1b* transgene^17^ to study responses to environmental estrogens. These transgenic zebrafish include an inserted green fluorescent protein (GFP) sequence and the expression of this reporter sequence is driven by ligand-receptor binding to either inserted or endogenous EREs. Alternative fluorescent reporter sequences to GFP used in transgenic (TG) models now include those that are photoconvertible such as the Kaede protein, where upon exposure to UV light, there is an irreversible spectral shift of the native (green) state from 508 nm (absorption) and 518 nm (emission) to longer wavelength peaks at 572 nm and 582 nm, respectively, resulting in a red state, comparable to the green state in terms of brightness and stability^36,37^. Application of photoconvertible proteins include for tracking individual cells during tissue development^38–40^.

In this study we generated a novel estrogen responsive transgenic zebrafish model with a Kaede photoconvertable (green to red) fluorescent protein (ERE-Kaede-Casper zebrafish) and applied it to assess for windows of tissue-sensitivity to estrogen exposure during early-life and to investigate how exposure to estrogen during early life affects responsiveness to environmental estrogens for subsequent exposures.

## Results

### ERE-Kaede-Casper model

**Figure 1:**
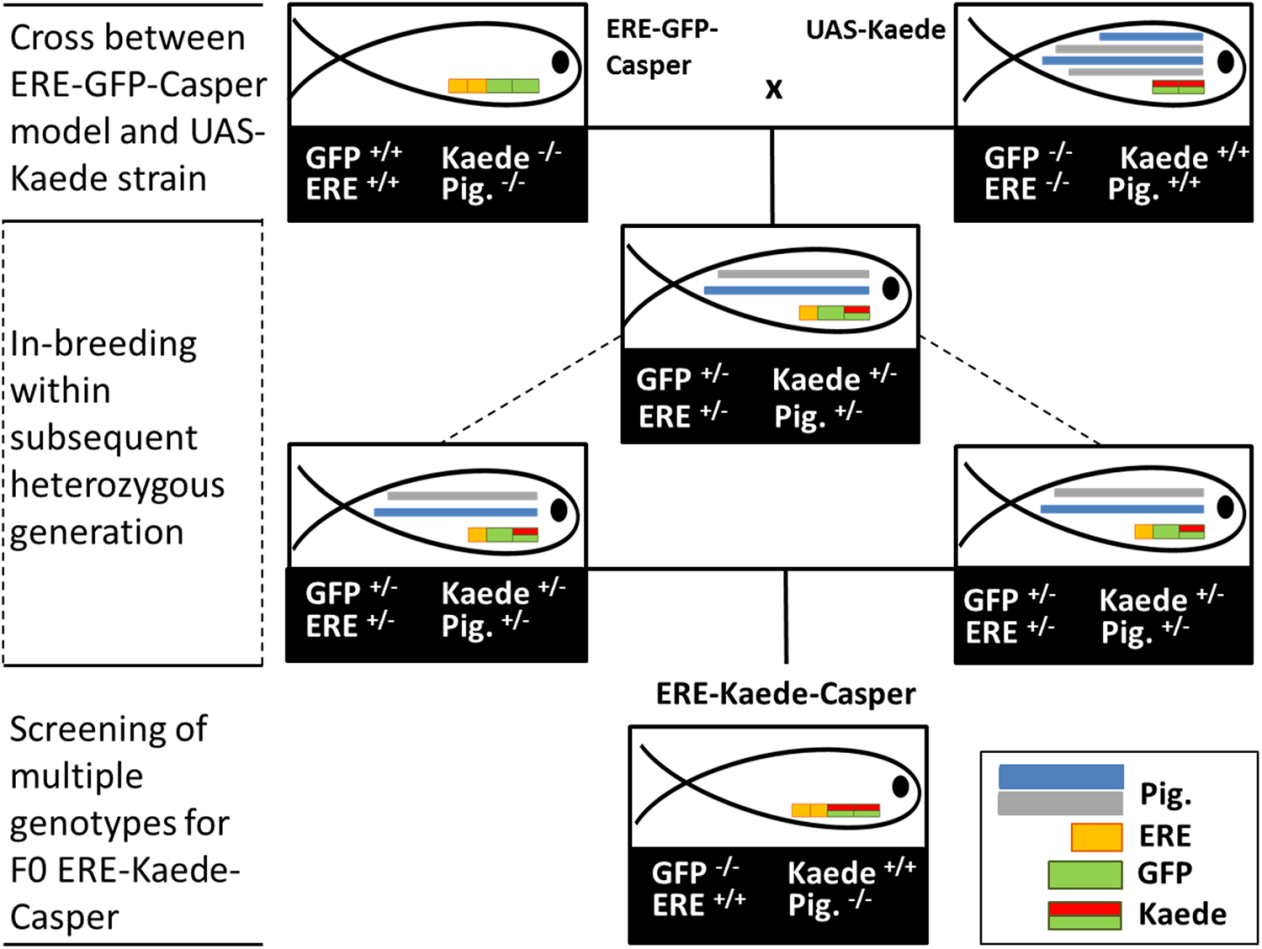
Generation of ERE-Kaede-Casper (F0) line. ERE denotes the ERE-Gal4ff transgene sequence, GFP denotes the UAS-GFP transgene sequence and Kaede denotes the UAS-Kaede transgene sequence. Expression of pigmentation (Pig.) genes roy (dark) and nacre (silver) are also shown. The ERE-GFP-Casper model, homozygous for both transgene sequences, and a homozygous UAS-Kaede strain were initially crossed to produce a heterozygous generation. In-breeding within this generation produced progeny with different genotypes based on four genes of interest. At sexual maturity, F0 ERE-Kaede-Casper adults were identified by screening for photoconvertible progeny with fully silenced pigmentation and TG(ERE:Gal4ff)(UAS:Kaede) expression.

A founder F0 generation of the ERE-Kaede-Casper model was established and a homozygous F1 generation generated and raised to adulthood for subsequent use for the exposure studies (Fig. 1). Tissue-specific responses in the ERE-Kaede-Casper model were consistent in subsequent generations for homozygous individuals as assessed via regular screening. Furthermore, there was high consistency in the response to estrogen exposure (tissue specificity and sensitivity) between the ERE-Kaede-Casper model and the original ERE-GFP-Casper model (Supplementary Fig. S2).

### Water Chemistry Analysis

In all water control samples chemicals were below the limit of quantitation (LOQ). For genistein, BPA, and EE2 measured concentrations at day 5 were highly consistent, at between 99% and 133% of nominals across the concentration ranges tested. Exposure concentrations are reported as ng/L or µg/L in the text but nM concentrations are included where direct comparisons between chemicals are made in both the text and in the figures. The full water chemistry analyses are provided in Supplementary Table S2.

### Tissue responses to EE2 during early life in the ERE-Kaede-Casper model

**Figure 2:**
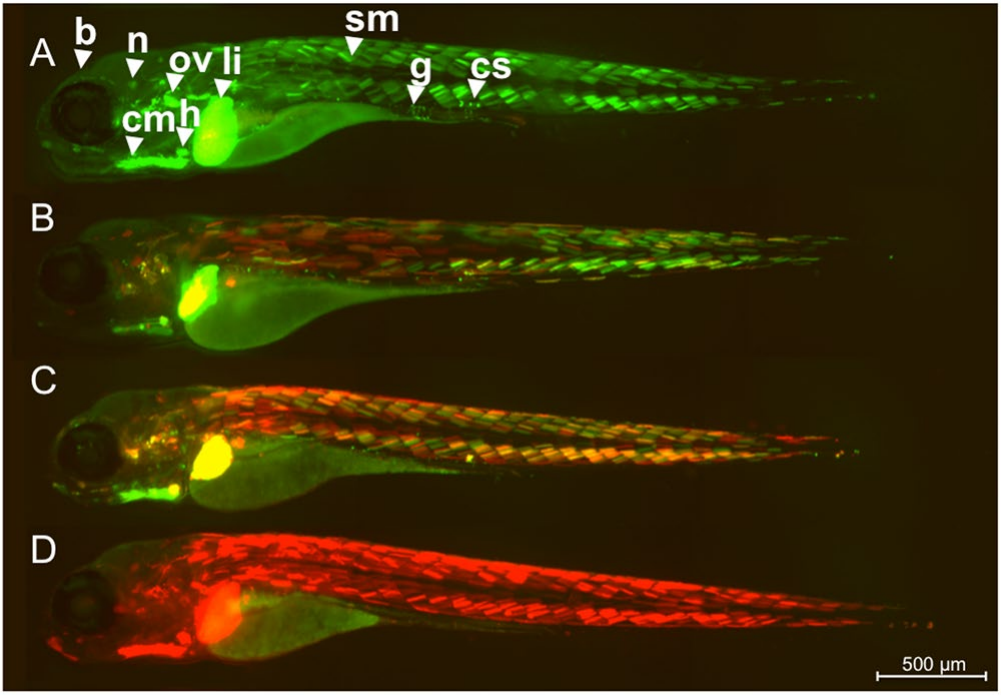
Kaede conversion analysis. ERE-Kaede-Casper larvae were exposed to 100 ng EE2/L over the period 0–5 dpf and imaged at 5 dpf either without UV exposure (**A**), or after exposure to UV at 3 dpf (**B**), 4 dpf (**C**) and 5 dpf (**D**) to convert Kaede fluorescence from green to red. Specific tissue response in the liver (li), heart (h), somite muscle (sm), otic vesicle (ov), cardiac muscle (cm), corpuscle of Stannius (cs), brain (b), neuromast (n), and gut (g).

Under UV illumination Kaede fluorescence was converted fully from green to red at the intervals tested over the life period 0–5 dpf (see Fig. 2D) thus enabling visualization and quantification of tissue responses to estrogen for multiple time windows and for repeat (see later) exposures in the same individual.

Exposure to EE2 induced a wide range of tissue responses during early life (0–5 dpf) in the ERE-Kaede-Casper model. Without photoconversion, tissues including liver, heart, gut, brain, somite muscle, corpuscle of Stannius and cranial muscle all showed high levels of fluorescence when imaged at 5 dpf after 100 ng EE2/L exposure (Fig. 2A). UV conversion of Kaede at 3 and 4 dpf, indicated differences in the temporal responses to EE2 stimulation for the different tissues. The heart and liver responded consistently to EE2 over the 0–5 day study period with new Kaede protein (green) expressed subsequent to UV photoconversion at 3 dpf and 4 dpf. Other tissues showed more variable temporal responses to EE2 during this period of development. Photoconversion highlighted different temporal expression of Kaede across regions of the tail. Muscle somites at the tip of the tail (caudal peduncle) showed a stronger response to EE2 between 3–5 dpf compared with the muscle somites nearer the abdomen, which appeared to become less responsive by 3 dpf (Fig. 2B). This difference in sensitivity can be seen more clearly after the 4 dpf photoconversion (Fig. 2C). Tissue surrounding the cranium appeared to be most responsive to EE2 after 4 dpf, with little or no Kaede expression before this time (no red fluorescence). The corpuscle of Stannius, a collection of cells located in the tail above the anus and involved in calcium homeostasis, responded most strongly to the EE2 treatment during 3–5 dpf. Preliminary data from our laboratory (not shown) suggest response in the brain to EE2 also appears to differ temporally for the early life exposures (Takesono *pers comm*).

### Protocol for investigating multiple estrogen exposures in the ERE-Kaede-Casper model

Tissue response patterns after the 48 h exposure to 10 ng EE2/L and 50 ng EE2/L were similar, but response intensity was positively associated with exposure concentration (Supplementary Fig. S3). Photoconvertion of the Kaede fluorescence after 24 h (at 3 dpf) and subsequent imaging demonstrated further delayed Kaede expression in liver and muscle somites for the 50 ng EE2/L treatment, but not for the 10 ng EE2/L treatment. Based on these findings, the protocol we adopted for priming with EE2 prior to subsequent exposure to environmental estrogens, was to expose embryo-larvae (0–48 hpf) to 10 ng EE2/L for 48 h followed by a 24 h incubation of the larvae in an estrogen-free embryo culture medium followed by photoconversion of the Kaede fluorescence via treatment with UV light for 2 minutes.

### Responses to environmental estrogens after early life exposure to EE2

**Figure 3:**
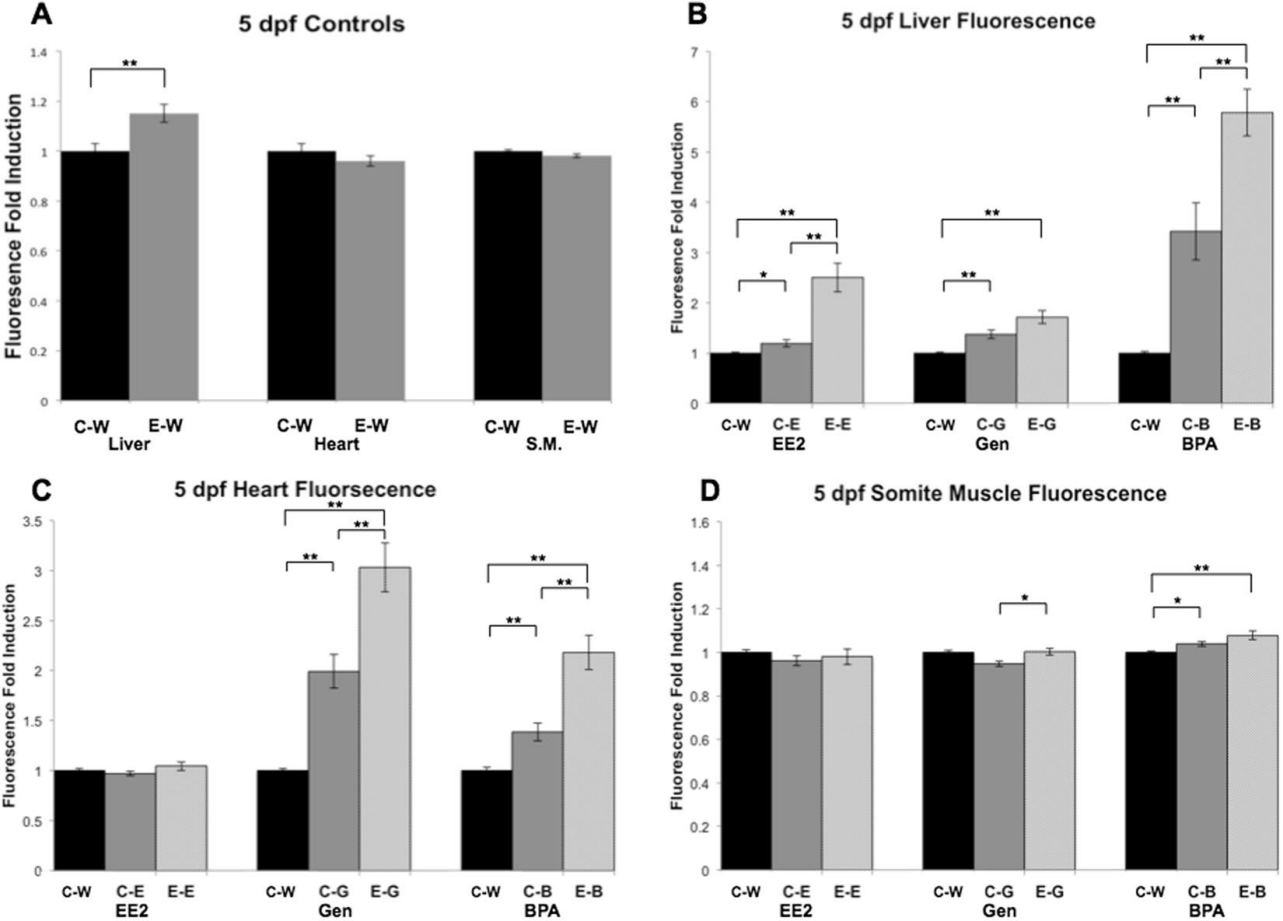
Quantification of target tissue responses in ERE-Kaede-Casper transgenic zebrafish exposed to estrogens during early life, as determined by fluorescence induction. Green fluorescence intensity was quantified in liver, heart and somite muscle (S.M.) in controls (**A**) at 5 dpf. Control (non-exposed) larvae and larvae exposed initially to 10 ng EE2/L over the period of 48 h (0–2 dpf) and green fluorescence intensity in liver (**B**), heart (**C**) and S.M. (**D**) were quantified after EE2 (10 ng/L), genistein (500 µg/L) and BPA (2000 µg/L) exposures for 3–5 dpf. Quantification of liver responses in the E-Chemical (E-E, E-G or E-B, respectively) treatment groups were normalized against their respective E-Water controls (**A**), which were set to a value of 1. Data are reported as mean fold induction ± SEM (n = 18). Statistical significance values were calculated using ANOVA and Games-Howell post-hoc test (*p < 0.05 and **p < 0.01).

Autofluorescence was detected in the yolk sac and otic vesicle only at 5, 7 and 11 dpf in control groups (C-Water and E-Water; Supplementary Information, Fig. S4), as has been shown to occur previously for the ERE-GFP-Casper model^32^. No green fluorescence was detected for the C-Water treated groups at 3, 5, 7 or 11 dpf, or for the E-Water controls, with the exception at 5 dpf where there was a 15% higher average pixel intensity in the liver (determined quantitatively by image analysis, Fig. 3A). Responses in the liver in the E-Chemical groups were thus normalized against the pixel intensity of the E-Water exposure for all time-points to account for the higher average pixel intensity in this tissue. Pixel intensity values for the heart and somite muscle in E-Water groups did not differ from the C-Water groups.

**Figure 4:**
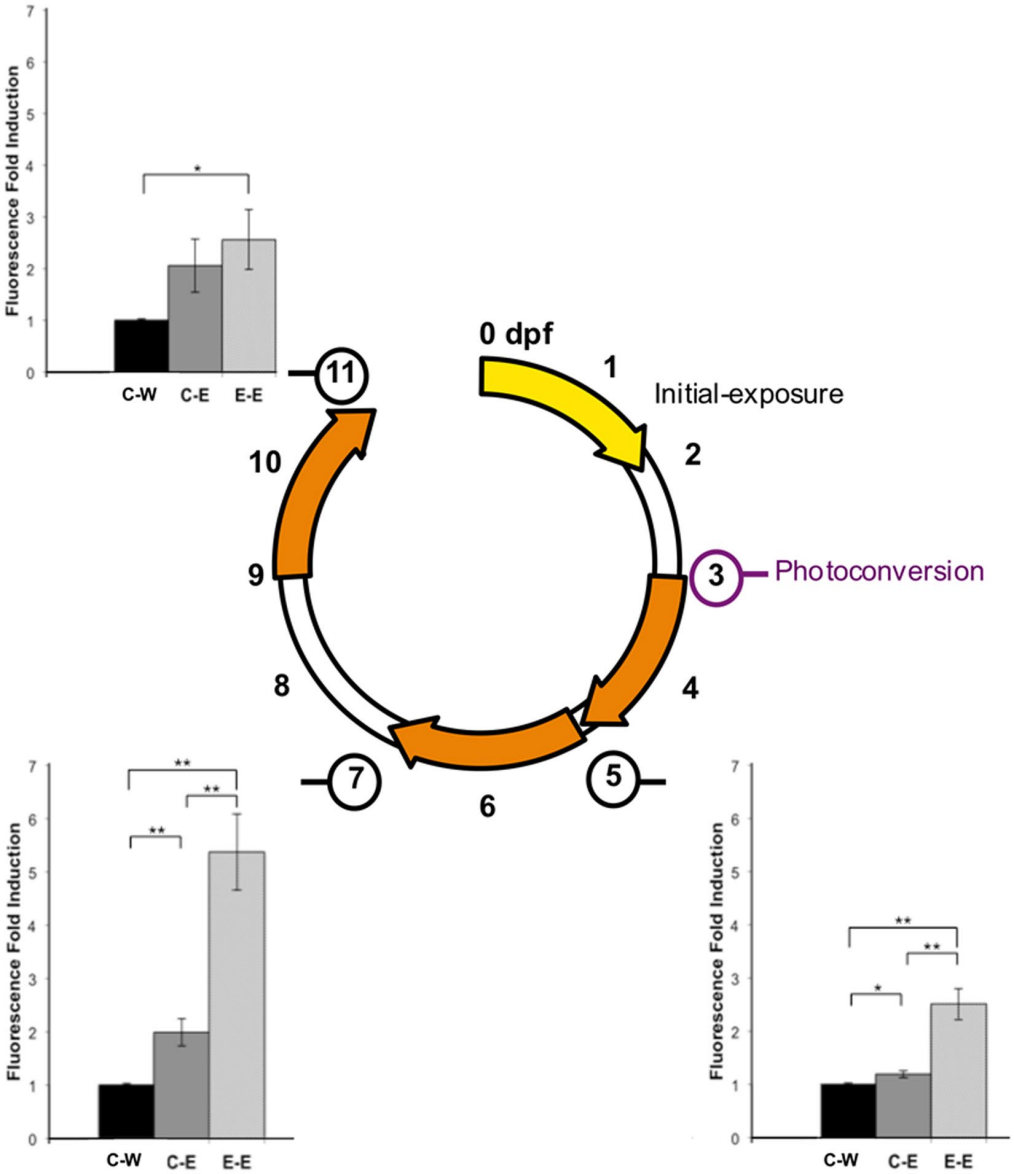
Quantification of liver responses in ERE-Kaede-Casper transgenic zebrafish exposed to EE2 at different stages of development, as determined by fluorescence induction. Responses in the liver were quantified after EE2 exposure at 3–5 dpf, 5–7 dpf and 9–11 dpf. Quantification of liver responses in the E-Chemical treatment groups were normalized against their respective controls. Data are reported as mean fold induction ± SEM (n = 18). Statistical significance values were calculated using ANOVA and Games-Howell post-hoc test (*p < 0.05 and **p < 0.01).

**Figure 5:**
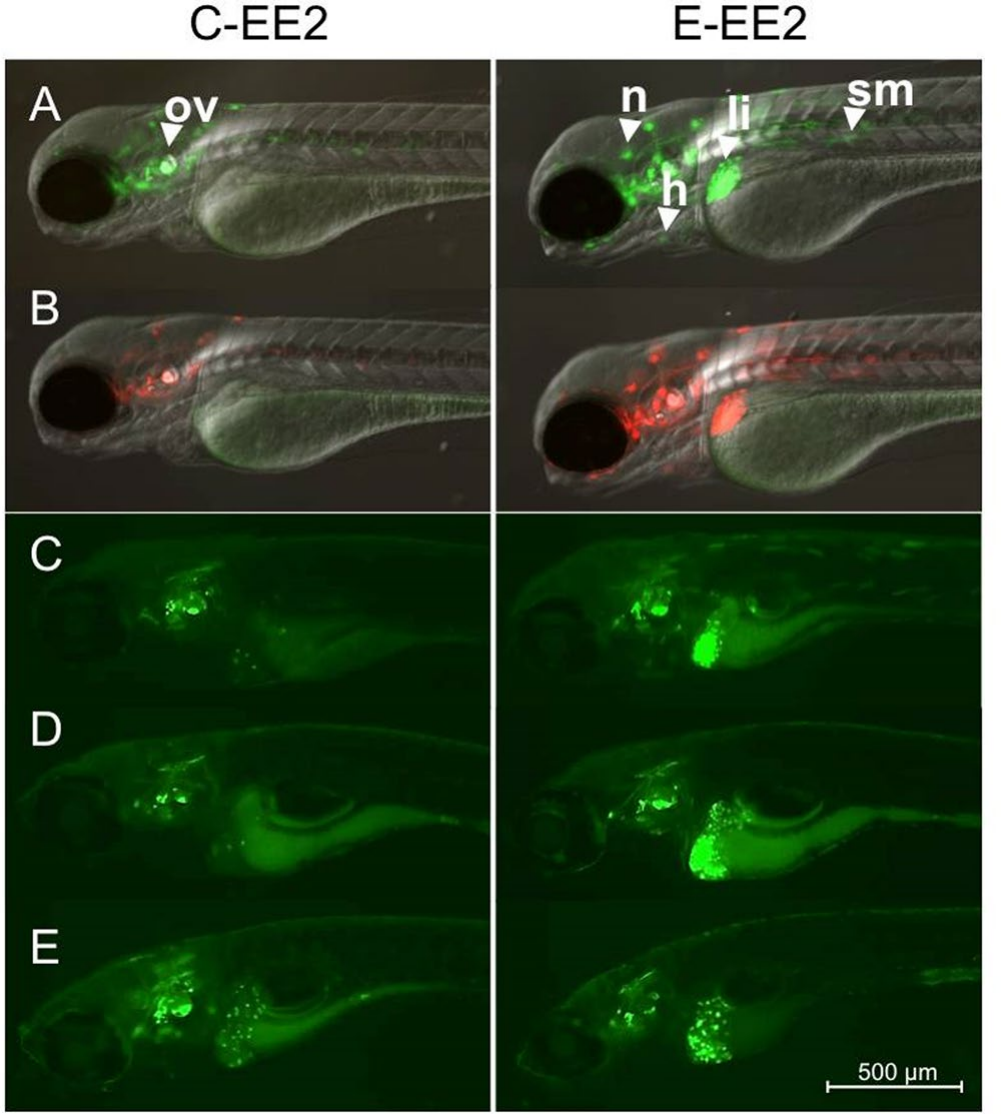
Sensitivity to ethinylestradiol for repeated exposures. Control (non-exposed) larvae and larvae exposed initially to 10 ng EE2/L over the period of 48 h (0–2 dpf) were imaged at 3 dpf (**A**) and the Kaede response was then converted fully from green to red fluorescence via UV exposure (**B**). Both groups of photoconverted larvae (control and EE2-exposed) were then exposed to 10 ng EE2/L over the period 3–5 dpf (**C**), 5–7 dpf (**D**) or 9–11 dpf (**E**) and imaged on the final day of exposure (n = 18). Newly generated Kaede expression (green fluorescence) in liver, heart and somite muscle green was quantified by image analysis. All images were acquired by inverted compound microscope using a 5× objective. (**A**) and (**B**) images were acquired using GFP, RFP and DIC filters. (**C**), (**D**), and (**E**) are presented with the GFP filter only. Specific tissue response in the liver (li), heart (h), somite muscle (sm), otic vesicle (ov) and neuromast (n).

Responses to the different estrogenic chemicals were highly consistent between individual embryo-larvae (Fig. 3). Exposure to EE2 during early life (0–48 hpf) affected subsequent responses to the exposures to EE2, BPA and genistein (3–5 dpf). In the liver at 5 dpf (3–5 dpf exposure) for exposure to EE2 (10 ng/L) and BPA (2000 µg/L) expression of GFP in E-Chemical groups was 682% and 98% higher than C-Chemical responses, respectively (Fig. 3B). This was also the case for responses in heart tissue at 5 dpf (3–5 dpf exposure), where responses to genistein and BPA were 105% and 206% higher respectively in primed E-Chemical groups than in unprimed C-Chemical groups (Fig. 3C). There was an apparent enhanced response to BPA in the somite muscle at 5 dpf, but the difference between C-BPA and E-BPA groups was not statistically significant (Fig. 3D). A small, but statistically significant difference, in somite muscle response occurred in the groups exposed to genistein (C-Gen and E-Gen) but neither of the groups’ fluorescence response was significantly higher compared with the C-Water control (Fig. 3D). There was higher fluorescence induction in the liver (342%) in the E-EE2 treatment compared with the C-EE2 groups for the exposures at 7 dpf (5–7 dpf exposure, Fig. 4), but no such difference between these treatment groups for the exposure at 11 dpf (9–11 dpf exposure, Fig. 4) indicating the enhanced responsiveness to estrogen may decay with time –i.e. for later life stages - in this issue. Fluorescence images for the quantified results (Fig. 4) are presented in Fig. 5.

### qPCR

Relative expression levels of the three ESRs (*esr1*, *esr2a* and *esr2b*) in whole bodies of ERE-Kaede-Casper zebrafish at 5 dpf after the exposures to EE2 (primary and a secondary exposures) are shown in Supplementary Fig. S5. For all three transcripts, expression appeared to be highest in the E-EE2 group, most notably for the *esr2b* gene, compared to C-Water larvae, but there were no statistically significant differences for the expression of any of the *esr*s between the different treatments.

## Discussion

We have generated a novel estrogen responsive transgenic model ERE-Kaede-Casper that has potential for studies into the effects of environmental estrogens, especially for studies considering life history exposure and interactive effects. Using the ERE-Kaede-Casper model we illustrate the dynamics of tissue responses to EE2 exposure, provide new information on the ontogeny of these responses and show enhancements in sensitivity in different body tissues for exposure to environmental estrogens following an initial exposure to EE2 during early life (0–2 dpf). The zebrafish model, generated by crossing two established transgenic models has a (high) sensitivity to estrogenic chemicals, comparable with our previously developed ERE-GFP-Casper model (Supplementary Fig. S2)^32^ and a silenced skin pigmentation that enhances fluorescence detection. We have shown that the Kaede chromophore can be successfully photoconverted in living intact individuals in all responding tissues and for high levels of Kaede expression, without any overt indication of development toxicity (Fig. 2). Translucency of the skin assisted efficiency of photoconversion as pigmentation normally blocks UV light penetration into the deeper tissues in larvae. The ability to photoconvert the Kaede fluorescence response in the ERE-Kaede-Casper model provides a more dynamic model for studies into temporal dynamics and mixture responses to estrogen compared with the ERE-GFP-Casper model. For the liver only, in some instances we found persistence of the green fluorophore of Kaede after applying two 1-minute UV light exposures. This may have been due to an incomplete conversion of the Kaede chromophore^37^ or as a consequence of the higher optical density and/or thickness of the liver, compared with some of the other responding body tissues (e.g. heart and somite muscle), that may also have limited UV penetrance and consequently inhibited the photoconversion process. However, this reduced Kaede photoconvertion efficiency in the liver of embryo-larval stages was easily accounted and adjusted for when calculating the response to estrogens in this tissue versus controls. It is likely that photoconversion efficiency in other body tissues may be reduced with further growth and development of the fish.

We show windows of sensitivity to EE2 for specific tissues during early development in the ERE-Kaede-Casper model. The heart and liver responded in a consistent manner to EE2 during the life period studied, between 0–5 dpf. In contrast, other tissues, including muscle somites and the brain, appeared to vary in their responses over this life period. The development of zebrafish tissues and organs has been studied extensively^41^ but the role and importance of estrogens in the development of individual somatic tissues is lacking. In mammals, estrogen has been shown to regulate growth and differentiation of a wide range of tissues including specific regions of the brain, bone, liver, and the cardiovascular system^42^. In zebrafish, studies have shown that phytoestrogens, such as genistein, can affect brain development when exposed during the early life-stage of growth^43^. Estrogen has recently been linked to cardiovascular maintenance and repair in zebrafish also^44^ and appears to play an important role in the development of the peripheral nervous system (PNS) within skeletal muscle^45^. These roles of estrogens are reflected in the tissue-specific responses observed in the ERE-Kaede-Casper model, and in other estrogen responsive transgenic zebrafish lines during early life-stages^32,46^.

The ERE-Kaede-Casper model was used to study tissue-specific responses following 0–2 dpf exposure to EE2. The results (Supplementary Fig. S3) show that fluorescence induction continued after the initial EE2 exposure for periods that varied depending on the exposure concentration. Kaede expression continued in the liver, heart, brain and somite muscle for 24 and 48 hours after exposure to 10 ng EE2/L and 50 ng EE2/L, respectively. Kaede expression was most prominent in the liver. This illustrated the ERE-Kaede-Casper model’s capability for studying temporal response dynamics to estrogenic chemicals exposures using photoconversion. The factors behind the different dynamics of response across the different responding body tissues over time are not known. They likely reflect variation in accumulation, metabolism and excretion of the chemical within these tissues, as well as possible differences in the number and types of ESRs that are expressed and dynamics concerning the conscription of cofactors. Zebrafish have been applied successfully for *in vivo* toxicokinetic studies assessing uptake, metabolism and excretion of estrogenic chemicals^47^. These are challenging studies however, as only small amounts of plasma can be obtained for analytical chemistry measurements placing major practical restrictions on what can be achieved studying the uptake dynamics of the chemical. The ERE-Kaede-Casper could provide a valuable model for supporting such toxicokinetic studies. The ability to photoconvert Kaede fluorescence could be applied as a proxy to assess for both the presence and persistence of the exposure chemical in the target tissues. This would operate on the assumptions that the level of Kaede expression is directly correlated with the parent chemical and that the products of metabolism are not biologically (estrogen) active. In many cases however, where the parent compound only is estrogen active the ERE-Kaede-Casper model could potentially offer an effective system to non-destructively study the toxicodynamics of estrogenic chemicals in zebrafish in real time.

There is a reliance on single chemical exposures for environmental effects assessments, but in contrast wildlife and humans are exposed intermittently, or continuously, to complex mixtures of chemicals, including EDCs. Many studies have now shown interactive (including additive) effects of estrogens and other EDCs^17,19^. Almost nothing, however, is known for the effects of repeated or sequential exposures to estrogens on tissue responses or on the health implications for these exposures, which will occur for many ambient environments^48^.

Here using the ERE-Kaede-Casper model, we show that exposure to EE2 during early life has a significant bearing on the subsequent responsiveness of body tissues to further estrogen exposure, but this responsiveness differs both for different estrogens - here for EE2, genistein and BPA, and the target tissue. For example, the liver appeared to be the most affected (sensitized) to EE2 after the initial early life exposure to EE2, where as the heart was the most responsive to genistein following an early life exposure to EE2. In support of our findings for genistein, the heart has been shown previously to be especially responsive to phytoestrogens, including genistein, in comparison to other tissues^32^ and has also been associated with adverse implications for cardiovascular maintenance and repair in zebrafish^44^. BPA has been linked to cardiovascular defects and abnormal liver enzymes in mammals^10,49^.

The mechanisms leading to the enhanced responsiveness of certain tissues, and not others, are not clear. Nor is it clear why this sensitization effect diminishes at later stages of development, as measured specifically in the liver in this study. Changes in ESR(s) number is proposed as a potential mechanism and is discussed further below. In addition, changes in response to estrogenic chemicals may have epigenetic origins via DNA methylation or histone acetylation of gene sequences (collectively known as the epigenome) related to estrogen signaling. Estrogen signaling genes are regulated, in part, through DNA methylation of their promoter regions in a gender- and region-specific manner^50–52^. Furthermore, DNA methylation and subsequently the transcription levels of ESR genes are influenced substantially by exposure to environmental chemicals at developmentally sensitive windows such as embryogenesis and early postnatal stages^53–55^. Although it is now widely accepted that chemicals affect the epigenome, epigenetic mechanisms are not yet considered in chemical risk assessment or utilized in the monitoring of the exposure and effects of chemicals and environmental change.

The expression of the ESR genes *esr1*, *esr2a* and *esr2b* was quantified in whole bodies using qPCR to investigate whether changes in receptor expression occurred for the different subtypes for the different treatment regimes (Supplementary Fig. S5). There was no change, however, in the expression of any of the subtypes across the different exposure groups. There was an indication that expression was higher for all ER subtypes in the E-E group treatment, but this was not statistically significant. In other studies, E2 (0.1 µM) has been shown to induce a significant increase in *esr1* expression after 96 h in zebrafish, using a similar exposure protocol and qPCR analysis^56^. Collectively, the findings suggest that changes in ESR(s) number may not be the major effect mechanism for the enhancement seen in the responses to environmental estrogens after an early life exposure to EE2. However, we say this with caution as measuring responses in whole body extracts is a relatively crude approach and tissue level effects analyses are needed to provide any degree of certainty on this assumption. Furthermore, as the qPCR analysis was conducted at 5 dpf and there may have been changes in the level(s) of *esr* expression prior to this analysis time-point that we could not account for (ER responses to estrogen have been shown to occur within 48 h in zebrafish)^56^. In summary, even with the above caveats we did not observe a clear trend in the *esr* expression dynamics that could be directly related to the sensitized responses to environmental estrogens caused by early life exposure to EE2.

In conclusion, we present a new ERE-Kaede-Casper zebrafish model incorporating a photoconvertible fluorescent protein that provides a novel approach for investigating the interactive effects of environmental estrogens *in vivo*, and studying biological responses for exposure scenarios that represent far more environmentally realistic scenarios that are studied currently. Applying this model we illustrate environmental risk assessment for estrogens needs to consider both the stage of development and exposure history of the organism as these factors affect the sensitivity and patterns of responsiveness to environmental estrogens.

## Methods

### Chemicals

17α-ethinylestradiol (EE2, CAS no. 57–63–6, ≥98% pure), genistein (Gen, CAS no. 446-72-0, ≥98% pure), a phytoestrogen and Bisphenol A (BPA, CAS no. 80-05-7, >99%pure)were used throughout this study.

### Animal Experiments

All animal work and experimental protocols used in this work were conducted in accordance with, and approved by, the University of Exeter’s Animal Welfare and Ethical Review Body, and undertaken under project and personnel licenses granted by the UK Home Office under the United Kingdom Animals (Scientific Procedures) Act.

### The ERE-Kaede-Casper Zebrafish Model

The ERE-GFP-Casper transgenic line was derived from an ERE-GFP-Casper line previously developed at the University of Exeter^32^ and a UAS-Kaede^57^ line from Max-Planck Institute of Neurobiology, Germany (Fig. 1). The ERE-GFP-Casper line is sensitive to estrogens, with GFP expression detected in hepatocytes for an exposure to 1 ng EE2/L, and shows tissue-specific responses to different estrogenic chemicals. The ERE-GFP-Casper line has silenced *roy* (dark) and *nacre* (silver) pigmentation genes (the “Casper” phenotype), resulting in a translucent phenotype and as a consequence improved GFP signal detection via fluorescence image analysis. The UAS-Kaede line has wild-type (WIK) pigmentation and expresses an inserted UAS-Kaede reporter transgene sequence. Details on the synthesis and testing of the new ERE-GFP-Casper transgenic line are provided in the Supplementary Information.

### Tissue responses to EE2 during early life in the ERE-Kaede-Casper model

We investigated tissue responses to EE2 for larval zebrafish between 0–5 days post fertilization (dpf) and the ability to photoconvert estrogen-induced green fluorescence in the Kaede-Casper model. ERE-Kaede-Casper larvae were exposed to 100 ng EE2/L over 0–5 dpf and exposed to UV light for 2 mins at the intervals of 3 dpf, 4 dpf and 5 dpf. A further group was exposed to 100 ng EE2/L over 0–5 dpf with no exposure to UV light. Larvae were then subjected to imaging at 5 dpf on an inverted compound microscope. After imaging, differential interference contrast (DIC), green and red Kaede fluorescence images were overlaid and the color of individual tissue response qualified via the ratios of green (new Kaede expression), red (‘old’ Kaede expression pre-photoconversion) and yellow (equal levels of new and old Kaede expression) fluorescence.

### Development of a Protocol for multiple estrogen exposures in ERE-Kaede-Casper model

To investigate for effects of estrogen exposure during early life on the subsequent responsiveness (sensitivity) to a further estrogen challenge we developed an experimental protocol to identify an appropriate exposure interval and concentration for the EE2 primary exposure. EE2 was adopted for these exposure studies because of its effects on a wide range of tissues in the ERE-GFP-Casper model, including at environmentally relevant concentrations^32^. The temporal dynamics of estrogen-induced fluorescence response was investigated for exposures to (nominal) 10 and 50 ng EE2/L. Twenty larvae were exposed to each of the two test EE2 concentrations and six larvae per concentration were imaged and subjected to photoconversion every 24 hours (2–5 dpf) to compare patterns and levels of new (green) and old (red) fluorescence induction at each time step.

### Quantifying responses to EE2 in the primary exposure

**Figure 6:**
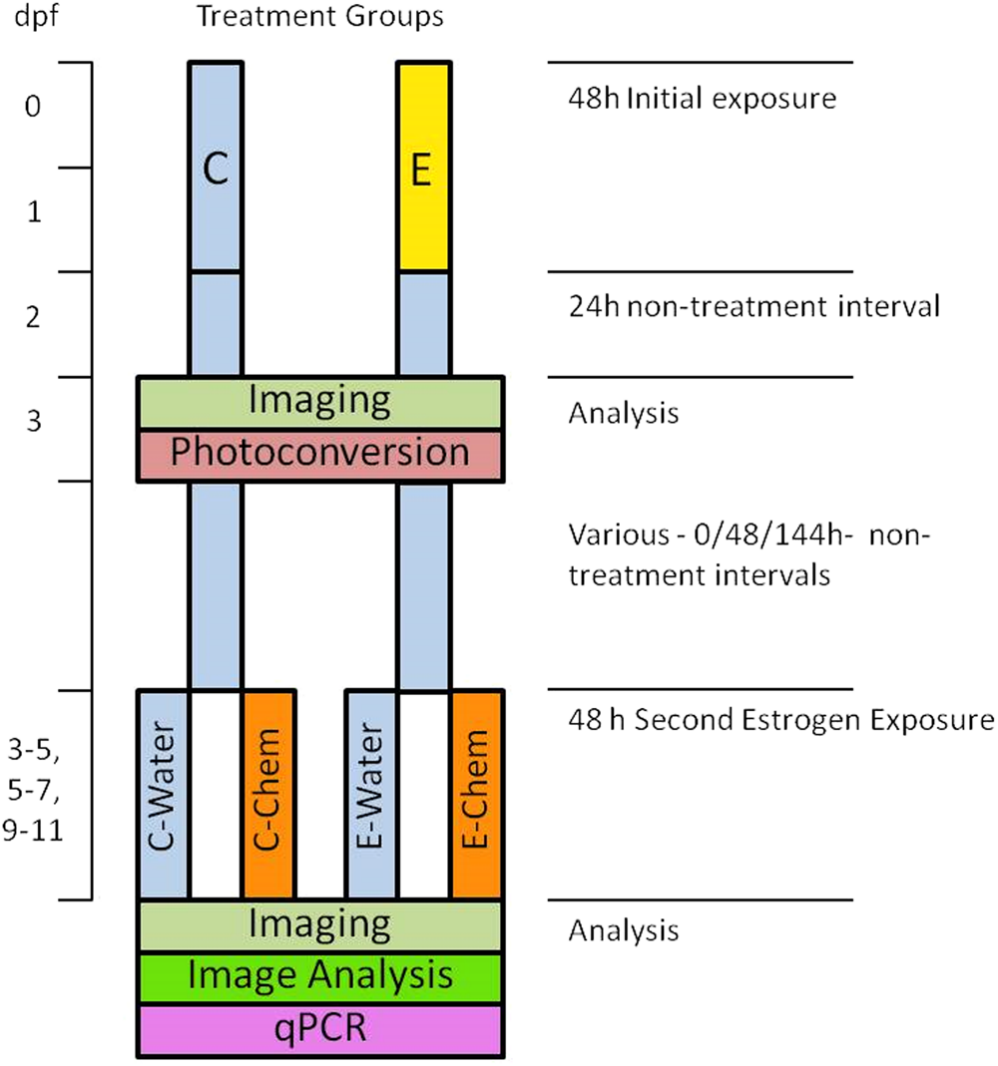
Exposure Protocol Outline. ERE-Kaede-Casper embryos were initially separated into 48 h control (**C**) and EE2 (10 ng/L) initial-exposure (**E**) groups. After a subsequent 24 h non-exposure period, larvae were imaged and Kaede expression underwent photoconvertion (green to red fluorescence, 3 dpf). Various intervals of non-exposure were then adopted before a second estrogen exposure was conducted. Larvae from the two initial treatments (**C** and **E**) were each divided into two groups; one control exposure (C-Water and E-Water) and the second an estrogenic chemical exposure (C-Chemical and E-Chemical). Imaging was carried out at the final time point with subsequent image analysis for quantification of Kaede expression. The expression of the three nuclear ESR subtypes was also quantified at the final time point using qPCR.

The experimental protocol for the multiple exposures studies is presented in Fig. 6. The initial exposure period was for 48 hours (0–2 dpf) to EE2 at a concentration of 10 ng/L. For the primary dosing to EE2, embryo-larvae (0–2 dpf) were cultured in embryo water either with (10 ng EE2/L, “E”) or without (0.1% final volume DMSO solvent control group, “C”) estrogen treatment. Using multi-well plates each treatment comprised of 6 wells containing 12 embryos (72 embryos per treatment). After the exposure larvae were removed from the incubation solutions, washed three times in embryo water and re-plated in their groups in estrogen (and solvent) free embryo water for a depuration period of 24 hours to allow for completion of Kaede expression in the estrogen treated larvae. At 3 dpf, 6 larvae from each well of the two treatment groups were imaged and all larvae were subjected to UV illumination to photoconvert any green fluorescence. Prior to imaging and UV illumination larvae were washed and anaesthetised in embryo water containing 0.008% tricaine, mounted in methylcellulose in embryo culture medium and placed into a glass bottom 35 mm dish (MatTek). Larvae were orientated to rest on their left side and images captured using an inverted compound microscope using GFP, RFP and DIC filters (1500 ms using filter set 38 HE: BP 470/40, FT 495, BP 525/50) with a 5 × objective. After imaging at 3 dpf, all larvae were mounted and exposed to 2 × 1 min bursts of UV light (DAPI filter) at 5 × magnification to fully convert the expressed Kaede to red fluorescence excitation and emission response wavelengths.

### Responses to environmental estrogens after early life exposure to EE2

Three estrogenic chemicals were chosen for the secondary exposures of the ERE-Kaede-Casper larvae, namely, EE2, BPA and genistein, all of which induce estrogen responses in different body tissues in zebrafish and have environmental relevance^32^. Single chemical concentrations were adopted for these studies: EE2 (10 ng/L), genistein (500 µg/L), BPA (2000 µg/L) and were based on activation of a low level of Kaede expression in the liver of the ERE-Kaede-Casper from initial screening trials (5 dpf larvae for a 48 h exposure) ensuring any potential increase or decrease in Kaede expression in the liver caused by EE2 pre-exposure would be both identifiable and quantifiable. Stock chemicals for each concentration were dissolved in analytical grade dimethyl sulfoxide (DMSO), stirred vigorously in glass vials for 24 hours, and stored at −20 °C. On the morning of exposure aliquots of stock solution were pipetted into 50 mL embryo culture water and stirred vigorously to give final nominal concentration working solutions (0.1% DMSO concentration).

ERE-Kaede-Casper larvae from the initial 48 h exposures (0.1% DMSO solvent control “C”, and EE2-exposed “E”) were subject to 24 h depuration subsequent to UV photoconversion and imaging, and at 3 dpf, (see Fig. 6) exposed to EE2, BPA or genistein. They were then incubated in estrogen (and solvent) free embryo medium for either 0, 48 or 144 hours (embryo water was changed every 24 h) prior to the second estrogen treatment. For these exposures, larvae were separated into four dosing groups; C-Water, C-Chemical, E-Water and E-Chemical (where Water denotes solvent control water, and Chemical is the second estrogen treatment – either EE2, BPA or genistein). ERE-GFP-Casper embryos (in embryo water) were pipetted into six-well plates, with twelve embryos per well. Each treatment regime consisted of 3 well replicates containing 12 larvae (36 larvae per treatment). The larvae were exposed to embryo water (Water) or estrogen treatment (Chemical) for a 48 h period. The exposure regimes were: EE2 3–5 dpf, 5–7 dpf and 9–11 dpf; BPA 3–5 dpf and genistein 3–5 dpf (Fig. 6). The imaging protocol was identical to that described for the first exposure studies (3 dpf stage for EE2) and was carried out at 5 dpf (EE2, BPA, genistein), 7 dpf (EE2), and 11 dpf (EE2). Images were collected for specific tissues, including the liver, heart and somite muscle, using a 10 × objective and green fluorescent Kaede expression quantified using ImageJ™ software. These tissues of interest were masked (outlined) manually to give a specific quantifiable region of interest (ROI) (Supplementary Fig. S1). The mean pixel intensity value from this ROI was used as a quantification of fluorescence response for the individual tissues.

### Analytical Chemistry

Two stock concentrations of each chemical were measured at 0 dpf and 5 dpf using tandem liquid chromatography-mass spectrometry (LC-MS), described in Green *et al*.^32^. For all chemicals, with the exception of EE2, water samples were diluted in acetonitrile (ACN) before analysis by LC-MS. Due to the low concentration of EE2, samples were initially concentrated using solid phase extraction (SPE) cartridges (Sep-Pak Plus C18) into ACN, to achieve a detectable concentration for LC-MS analysis (see Green *et al*.^32^, Supplementary Information for full protocol and results).

### qPCR

Relative expression levels of the three ESRs (*esr1*, *esr2a* and *esr2b*) in whole bodies of ERE-Kaede Casper zebrafish were analyzed using quantitative polymerase chain reaction RT-qPCR at 5 dpf after the exposures to EE2 (primary and a secondary exposure). Efficiency-corrected relative expression levels^58^ were determined by normalizing to the expression levels of the reference gene ribosomal protein L8 (*rpl8*) measured in each sample. For full details of the qPCR protocol see Supplementary Information, details on primer sequences, sizes of PCR products and PCR assay conditions are provided in Supplementary Table S1.

### Statistical Analysis

For the imaging data in the definitive estrogen exposure studies tissue-specific intensity values from the four treatment groups C-Water, E-Water, C-Chemical and E-Chemical were converted to a fold- increase value over their respective controls (C-Water repeat average intensity value). Tissue specific percentage-increases for the three repeats for each treatment group (6 replicates for each treatment, repeated 3 times, final n = 18) were averaged to give a single fold-increase value per treatment group. All values are presented as mean ± SEM. Statistical significance between treatment groups is indicated at the p < 0.05(*) or < 0.01(**) level, calculated using an ANOVA and Games-Howell post-hoc test. Using mean fold-increase data, responses from the E-Chemical groups were compared to C-Chemical groups and presented as percentage-increase values in the text, so as to differentiate from fold-increase over C-Water values. The two control groups (C-Water and E-Water) that were incubated in embryo water during the second exposure period were expected to produce no new (green) fluorescence response in tissues after the second exposure period. However, it could not be assumed that there would be complete Kaede photoconversion (green to red fluorescence) by UV light following the initial exposure period. Therefore, if the pre-exposed control group (E-Water) showed a statistically significant fold-increase to the equivalent C-Water control tissue value, the other pre-exposed group (E-Chemical) results were then normalized based on this fold-increase on the assumption that green fluorescence had remained after incomplete photoconversion at the 3 dpf stage.

After qPCR analysis, relative *esr* subtype expression values from the four treatment groups C-Water, E-Water, C-Chemical and E-Chemical were quantified in terms of increased level of expression above their respective control (C-Water repeat average value). *esr* subtype percentage-increases for the three replicates (final n = 3) for each treatment group were then averaged to give a single fold-increase value per treatment group. All values presented as mean ± SEM and statistical significance was calculated using an ANOVA.

### Data availability

All data generated or analyzed during this study are included in this published article (and its Supplementary Information files).

## Electronic supplementary material

Supplementary Information
