# Supplementary material for: Early life exposure to ethinylestradiol enhances subsequent responses to environmental estrogens measured in a novel transgenic zebrafish
Source: Sci Rep. 2018 Feb 9;8:2699. doi: 10.1038/s41598-018-20922-z (PMC5807302; doi:10.1038/s41598-018-20922-z)
Supplement: Supplementary file 1 — Supplementary Information [file 41598_2018_20922_MOESM1_ESM.pdf]

Supplementary Information

Early life exposure to ethinylestradiol enhances subsequent responses to environmental estrogens measured in a novel transgenic zebrafish

Jon M. Green[1], *Anke Lange*[1], *Aaron Scott*[1], *Maciej Trznadel*[1], *Htoo Aung Wai*[1], *Aya Takesono*[1], *A. Ross Brown*[1], *Stewart F. Owen*[2], *Tetsuhiro Kudoh*[1], *Charles R. Tyler*[1*]

[1]Biosciences, College of Life and Environmental Sciences, University of Exeter, Geoffrey Pope, Stocker Road, Exeter, Devon, EX4 4QD, UK.

[2] AstraZeneca, Global Environment, Alderley Park, Macclesfield, Cheshire, SK10 4TF, UK

*Corresponding Author: Charles R. Tyler E-mail: C.R.Tyler@exeter.ac.uk

Summary: Nine pages containing information on generating the ERE-Kaede-Casper zebrafish model, the image analysis protocol (Supplementary Fig. S1); the qPCR protocol (Supplementary Table S1); results from water chemistry analyses (Supplementary Table S2); results from model comparison (Supplementary Fig. S2); results from temporal analysis of fluorescence induction (Supplementary Fig. S3); Kaede expression in control groups at 5, 7 and 11 dpf (Supplementary Fig. S4); results of ER expression from qPCR analysis (Supplementary Fig. S5).

Generation of the ERE-Kaede-Casper Zebrafish Model As detailed in the main text, the ERE-GFP-Casper transgenic line was produced from crossings of an ERE-GFP-Casper line previously developed at the University of Exeter (Green et al 2016), and a UAS-Kaede52 line from Max-Planck Institute of Neurobiology, Germany. To enhance both the transmission rate of transgenic sequences to subsequent generations and consistency in estrogen response sensitivity, the ERE-GFP-Casper model was initially screened for homozygous adults by pair breeding individuals with wild-type (WIK strain) adults and assessing the ratio of fluorescent/non-fluorescent offspring produced (homozygous ERE-GFP-Casper adults produce 100% fluorescent offspring whereas heterozygous ERE-GFP-Casper adults produce 75% or a lower proportion of fluorescent offspring). Homozygous ERE-GFP-Casper adults were then crossed with the UAS-Kaede line in 10 different mating pairs, rotating the pair combinations to promote genetic diversity. Progeny from these crosses were heterozygous for five genes of interest; ERE:Gal4ff, UAS:GFP and UAS:Kaede transgene sequences and silenced roy and nacre pigmentation genes. Crossing of progeny from distinct families produced larvae with different combinations of the genes of interest. These larvae were screened for the homozygous ERE-Fluorescence and Casper genotype via exposure to 50 ng EE2/L from 1-3 days post fertilisation (dpf). Individuals carrying both Casper phenotype and fluorescence expression, as determined by their response to 50 ng EE2/L, were raised to sexual maturity. Screening for Kaede expression was not carried out in this first screen due to potential developmental effects of UV exposure required for distinguishing the protein from GFP. At sexual maturity, these fish were screened for Kaede expression via exposure to 50 ng EE2/L for 5 days (from 0-5 dpf) and a 2 min UV exposure to convert the Kaede excitation and emission response. Adults with progeny exclusively expressing Kaede protein (complete conversion from green to red fluorescence upon UV exposure) were

identified as the founder generation (F0) of the ERE-Kaede-Casper line. These fish were then bred to generate an F1 generation and sexually mature F1 fish were screened for homozygous expression of the transgene sequences as described for F0. We compared responses to EE2 in the ERE-Kaede-Casper line with our original ERE-GFP-Casper model to assess for sensitivity and tissue response patterning to estrogenic chemicals. To do so, embryos from both models were exposed to 10 ng EE2/L from 0-5 dpf and fluorescence response was observed using an inverted compound microscope (Zeiss Axio Observer) under consistent GFP excitation (180 ms using filter set 38 HE: BP 470/40, FT 495, BP 525/50). Further generations have been established for the ERE-Kaede-Casper line, and each generation has been screened to confirm that only homozygous adults are used for ensuring consistently high sensitivity.

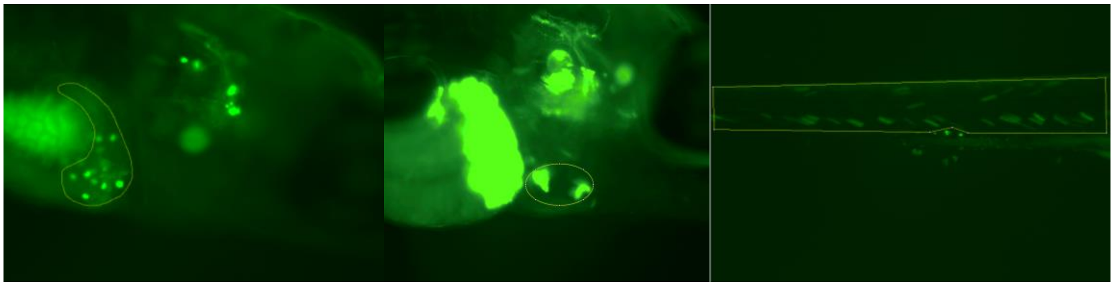

Figure S1. **Image analysis**. Fluorescence induction for specific tissues was quantified via initially manually masking that tissue (see: yellow outline for liver (A), heart (B) and somite muscle (C) and quantified using ImageJ™ software.

qPCR Protocol Effects of the initial exposure to EE2 were assessed on the expression of esr1, esr2a and esr2b for the secondary EE2 exposure via qPCR. Three pools of 15 embryos from the secondary exposure to EE2 (at the 3-5 dpf stage) were collected from each treatment regime after imaging at 5 dpf, snap frozen in liquid nitrogen and stored at -80 °C until analysis. Total RNA was extracted using Tri reagent (Sigma-Aldrich, UK) according to the manufacturer's protocol. The quantity and quality of the RNA was assessed spectrometrically using a NanoDrop 1000 Spectrophotometer. Two µg total RNA were treated with RQ1 RNase-Free DNase (Promega, Southampton, UK) and subsequently reverse transcribed into cDNA using M-MLV Reverse Transcriptase (Promega) and random hexamers (Eurofins Genomics), following manufacturer's instructions. The expression of target mRNA was subsequently determined by qPCR using target-specific SybrGreen assays on a CFX96 Real-Time PCR Detection System (Bio-Rad Laboratories Ltd., Hercules, CA, US) followed by melt curve analysis to validate amplification of a single PCR product. Efficiency-corrected relative expression levels were determined by normalising to the reference gene ribosomal protein L8 (rpl8), which was measured in each sample. rpl8 has been shown to be expressed at a consistent level across tissue types and experimental conditions (Filby & Tyler 2007), including after exposure to a wide range of chemicals and stressors in fish (e.g.: in zebrafish exposed to the organophosphorus flame retardant tris(1,3-dichloro-2-propyl) phosphate (Wang et al. 2015), the thyroid disruptor Di-(2-ethylhexyl) phthalate (DEHP) (Jia et al. 2016) and to silver (van Aerle et al. 2013) as well as to EE2 in adult Mangrove Rivulus (Farmer et al. 2012) and to hypoxia in fathead minnow (Hala et al 2012)). Details on primer sequences, sizes of PCR products and PCR assay conditions are provided in Table S1.

Gene name	gene symbol	sense primer	antisense primer	Product size (bp)	Ta (°C)	E[2]
estrogen receptor 1	*esr1*	CGAGTGCCGCTGTATGAC	TGCTGCTGCTGGTTGTG	130	59.5	1.97
estrogen receptor 2a	*esr2a*	AGGAGAAAACCAAGTAAACCAATC	AGGCTGCTAACAAGGCTAATG	173	59.0	1.91
estrogen receptor 2b	*esr2b*	TGAGGAGATGGTGAACAAGAC	ACTGATGGATGGATGAATGAAATG	132	57.8	2.03
ribosomal protein 18	*rpl8*	CCGAGACCAAGAAATCCAGAG	CCAGCAACAACACCAACAAC	91	59.5	2.09

[1] primers sequences from Filby et al. (2010) [2] E = PCR efficiency

Table S1. Target genes, primer sequences[1], amplicon size, annealing temperature and PCR efficiency for the RT-QPCR assays used in this study.

	EE2	Genistein		BPA	
**LOQ (µg/L)**	**0.0078**	**1.0**		**1.0**	
**Nominal (µg/L)**	**0.1**	**6.25**	**1000**	**125**	**2000**
**Day 0**	**0.14* (140%)**	**58.6 (94%)**	**1001 (100%)**	**137.5 (115%)**	**2200 (110%)**
**Day 5**	**0.099 (99%)**	**62.1 (99%)**	**956 (96%)**	**132.4 (103%)**	**2669.14 (133%)**

Table S2. Measured chemical concentrations in the embryo incubation water.

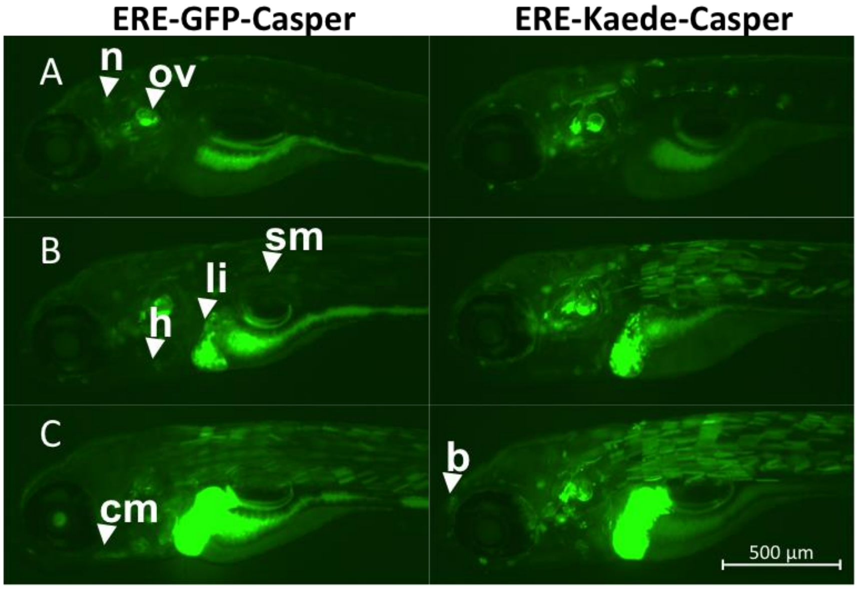

Figure S2: Comparison of ERE-GFP-Casper and ERE-Kaede-Casper models. Sensitivity and tissue response of the two models were compared via imaging after 0-5 dpf exposures to embryo water (A), 5 ng/L EE2 (B) and 10 ng/L EE2 (C). All images were acquired by inverted compound microscope (Zeiss Axio Observer) using a 5x objective. Images are presented with the GFP filter only.

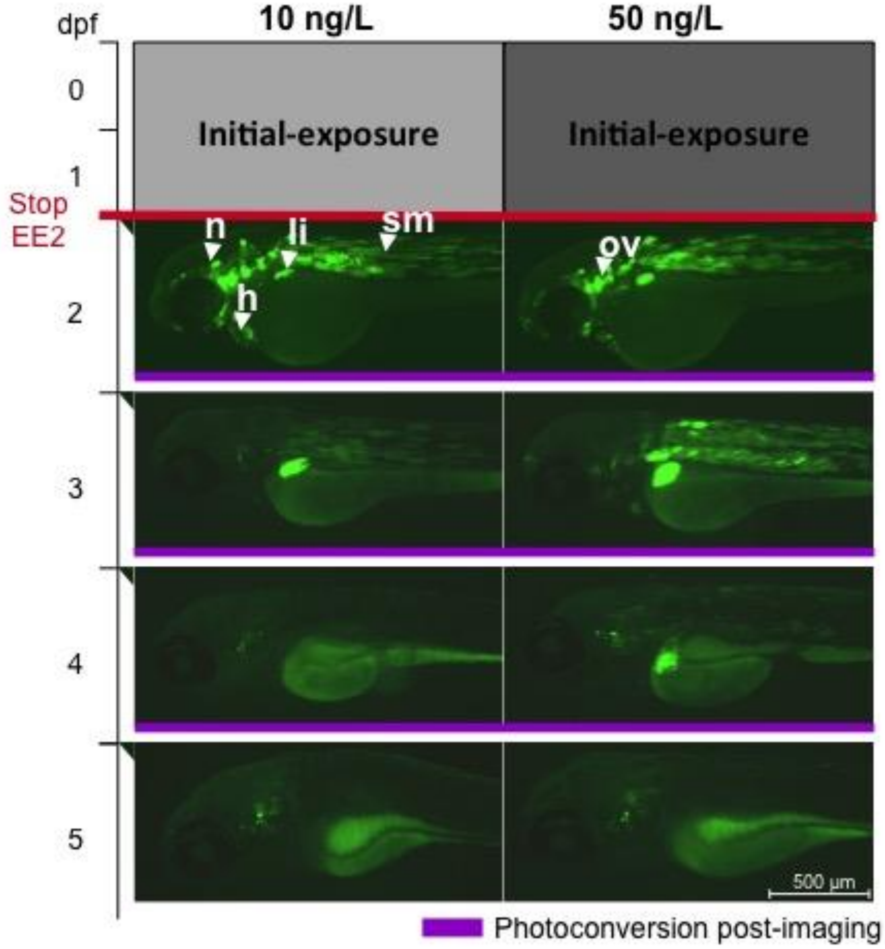

Figure S3. Temporal analysis of fluorescence induction after a single 48h exposure exposure to EE2. ERE-Kaede-Casper embryos were exposed to 10 ng/L and 50ng/L EE2 for 48

hours then washed and incubated in embryo water up to 5 dpf. At intervening 24 hour time-points larvae were imaged to assess new Kaede expression, then immediately exposed to UV light to photoconvert Kaede fluorescence from green to red. All images were acquired by inverted compound microscope (Zeiss Axio Observer) using a 5x objective. Images are presented with the GFP filter only and therefore only 24 hours worth of Kaede expression is shown in each image.

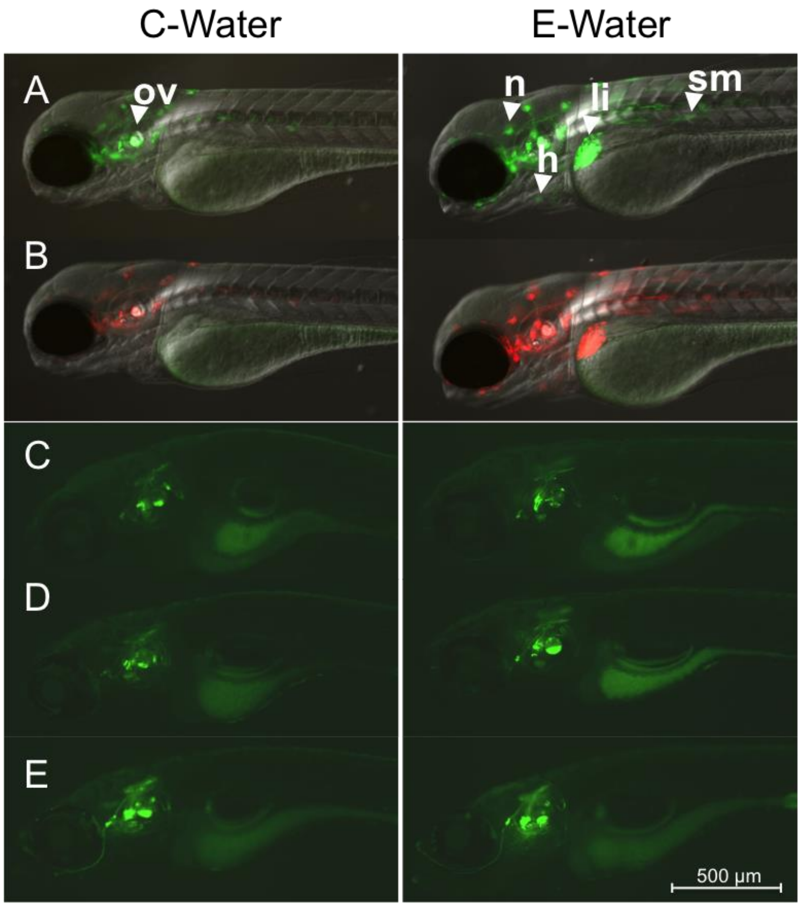

Figure S4. Kaede expression in control groups C-Water and E-Water. Control (non-exposed) larvae and larvae that were exposed initially to 10 ng EE2/L over the period 0-2 dpf

were imaged at 3 dpf (A) and the Kaede response then converted fully from green to red fluorescence via UV exposure (B). Both groups of larvae (control and EE2-exposed) were then exposed to embryo water over the period 3-5 dpf (C), 5-7 dpf (D) or 9-11 dpf (E) and imaged on final day of exposure (n=18). Newly generated Kaede expression (green fluorescence) was not observed (there was some auto-fluorescence). All images were acquired by inverted compound microscope (Zeiss Axio Observer) using a 5x objective. A and B images were acquired using GFP, RFP and DIC filters. C, D, and E are presented with the GFP filter only.

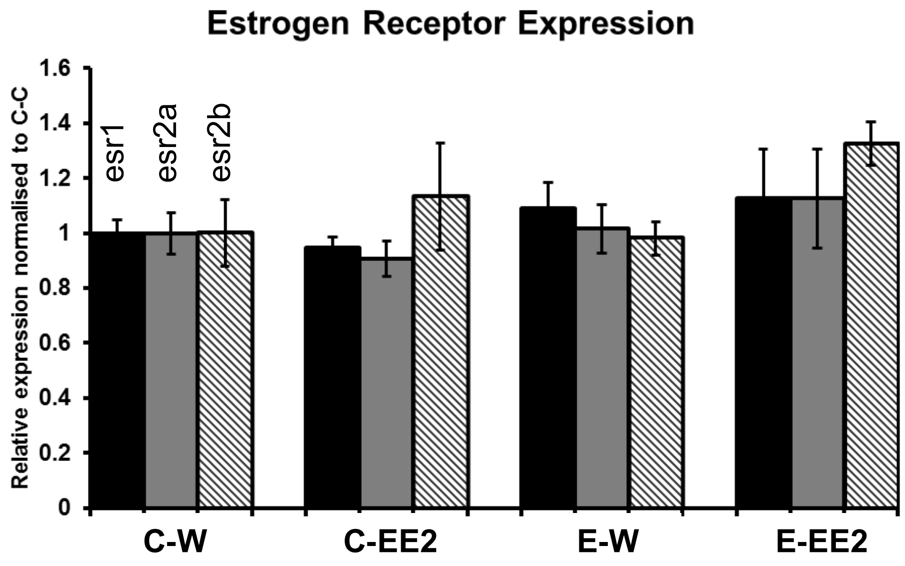

Figure S5: qPCR quantification of esr1, esr2a and esr2b gene expression. Results from qPCR analysis of esr gene expression in the four treatment groups from the 3-5 dpf second EE2 exposure treatment. Larvae were sampled at 5 dpf. Expression of the individual esr genes were compared for each treatment group. Data are reported as mean fold-induction ± SEM (n=3).

REFERENCES

Filby, A. L. & Tyler, C. R. Appropriate 'housekeeping' genes for use in expression profiling the effects of environmental estrogens in fish. *BMC Molecular Biology*, **8**, 10 (2007). doi: 10.1186/1471-2199-8-10

Farmer, J. L. & Orlando, E. F. Creating Females? Developmental Effects of 17α-Ethynylestradiol on the Mangrove Rivulus' Ovotestis. *Integrative and Comparative Biology*, **52**, 769–780 (2012). doi: 10.1093/icb/ics110

Wang, Q., Lam, J. C., Han, J., Wang, X., Guo, Y., Lam, P. K., & Zhou, B. Developmental exposure to the organophosphorus flame retardant tris(1,3-dichloro-2-propyl) phosphate: Estrogenic activity, endocrinedisruption and reproductive effects on zebrafish. *Aquatic Toxicology*, **160**, 163–171 (2015). doi: 10.1016/j.aquatox.2015.01.014

Jia, P. P., Ma, Y. B., Lu, C. J., Mirza, Z., Zhang, W., Jia, Y. F., Li, W. G. & Pei, D. S. The effects of disturbance on hypothalamus-pituitary-thyroid (HPT) axis in zebrafish larvae after exposure to DEHP. *PLoS ONE* 11(5): e0155762 (2016). DOI: 10.1371/journal.pone.0155762

Hala, D., Petersen, L. H., Martinovic, D. & Huggett, D. B. Constraints-based stoichiometric analysis of hypoxic stress on steroidogenesis in fathead minnows, *Pimephales promelas*. *Journal of Experimental Biology*, **215**, 1753-1765 (2012). doi: 10.1242/jeb.066027

van Aerle, R., Lange, A., Moorhouse, A., Paszkiewicz, K., Ball, K., Johnston, B. D., de-Bastos, E., Booth, T., Tyler, C. R., Santos, E. M. Molecular mechanisms of toxicity of silver nanoparticles in zebrafish embryos. *Environmental Science & Technology* **47**, 8005– 8014 (2013). doi: 10.1021/es401758d

Filby, A. L., Paull, G. C., Hickmore, T. F., & Tyler, C. R. Unravelling the neurophysiological basis of aggression in a fish model. *BMC Genomics* **11**, 498 (2010), doi: 10.1186/1471-2164-11-498
